# Bovine Lactoferrin Inhibits Dengue Virus Infectivity by Interacting with Heparan Sulfate, Low-Density Lipoprotein Receptor, and DC-SIGN

**DOI:** 10.3390/ijms18091957

**Published:** 2017-09-12

**Authors:** Jo-Mei Chen, Yi-Chin Fan, Jen-Wei Lin, Yi-Ying Chen, Wei-Li Hsu, Shyan-Song Chiou

**Affiliations:** Graduate Institute of Microbiology and Public Health, College of Veterinary Medicine, National Chung Hsing University, Taichung 40227, Taiwan; chenjm0317@gmail.com (J.-M.C.); yichinfan0309@gmail.com (Y.-C.F.); provista.wei@gmail.com (J.-W.L.); irisgf1986@gmail.com (Y.-Y.C.); wlhsu@dragon.nchu.edu.tw (W.-L.H.)

**Keywords:** dengue virus, bovine lactoferrin, heparan sulfate, low-density lipoprotein receptor, DC-SIGN

## Abstract

Bovine lactoferrin (bLF) presents in milk and has been shown to inhibit several viral infections. Effective drugs are unavailable for the treatment of dengue virus (DENV) infection. In this study, we evaluated the antiviral effect of bLF against DENV infection in vivo and in vitro. Bovine LF significantly inhibited the infection of the four serotypes of DENV in Vero cells. In the time-of-drug addition test, DENV-2 infection was remarkably inhibited when bLF was added during or prior to the occurrence of virus attachment. We also revealed that bovine LF blocks binding between DENV-2 and the cellular membrane by interacting with heparan sulfate (HS), dendritic cell-specific intercellular adhesion molecule 3-grabbing non-integrin (DC-SIGN), and low-density lipoprotein receptors (LDLR). In addition, bLF inhibits DENV-2 infection and decreases morbidity in a suckling mouse challenge model. This study supports the finding that bLF may inhibit DENV infection by binding to the potential DENV receptors.

## 1. Introduction

Lactoferrin (LF) is an iron-binding glycoprotein of 692 amino acids in length, and is present in breast milk and mucous secretions [1]. LF is a multifunctional protein that is involved in iron transportation in the intestines and blood, the inhibition of viral and bacterial infections, the modulation of immunity, and nonspecific immune responses [1,2]. The excellent antiviral activity of LF has been shown to prevent several viral infections, particularly ones caused by intestinally and mucosally-transmitted viruses, by binding to viral particles or viral receptors on the host cell membrane [3,4]. Positively-charged LF can interact with negatively-charged compounds such as glycosaminoglycan (GAG), thus inhibiting virus-receptor interaction [5]. In addition to interacting with GAGs, LF prevents viral infections by binding to dendritic cell-specific intercellular adhesion molecule 3-grabbing non-integrin (DC-SIGN) and low-density lipoprotein receptors (LDLR) [6,7].

Dengue is an important mosquito-borne viral disease in tropical and subtropical regions. The responsible pathogen, dengue virus (DENV), consists of four distinctive serotypes: DENV-1, -2, -3, and -4. The infection can be mild or can result in serious clinical presentations, causing the mild dengue fever (DF), the more serious dengue hemorrhagic fever (DHF), or dengue shock syndrome (DSS) [8]. DENV is an encapsidated and enveloped virus consisting of genome of 11 kb positive-sense, single-stranded RNA that encodes 10 viral proteins, including a capsid, a premembrane/membrane, and an envelope (E) protein; and seven nonstructural proteins, designated NS1, NS2A, NS2B, NS3, NS4A, NS4B, and NS5 [9].

The initial step of viral infection is the most critical: the interaction and subsequent binding between virions and viral receptor(s) on the host cellular membrane [10]. The DENV E protein has been shown to interact with GAG, DC-SIGN, and other possible receptors on the host cellular membrane [11,12]. Highly sulfated GAG, heparan sulfate (HS), and multi-sulfur chemicals (e.g., suramin), interact with the amino acid residues 284 to 310 and 386 to 411 of the DENV E protein, and inhibit DENV infection [11]. Thus, it has been suggested that the interaction of the dengue virus E protein with highly sulfated GAG on host cells is an important determinant for virus infection [11]. The C-type transmembrane receptor DC-SIGN has also been shown to be a prominent DENV receptor [13]. The DENV uses DC-SIGN as a receptor to infect human dendritic cells [12]. Several carbohydrate-binding compounds, such as mannose-specific plant lectins from Hippeastrum hybrids (HHA), have been shown to block DENV infection by binding either to the virus or to DC-SIGN [14,15].

Mosquito-borne viruses including Sindbis virus and Semliki Forest virus were inhibited by a LF–HS interaction [16]. Our previous study has shown that bovine lactoferrin (bLF) blocks Japanese encephalitis virus (JEV) infection by binding to HS and LDLR, but not by binding to virus particles [6]. Effective antiviral drugs are currently unavailable for treating DENV infection. In this study, we investigate the antiviral activity of bLF against DENV in vitro and in vivo. The results show that bLF blocks DENV-2 entry into cells by binding to HS, DC-SIGN, and LDLR, decreasing viral replication, and resulting in a reduction of morbidity in a mouse model.

## 2. Results

### 2.1. Bovine LF Blocks DENV Infection

In order to study the anti-DENV activity of bLF, Vero cell monolayers were infected with DENV-1 to -4 at a multiplicity of infection (MOI) of five for 24 h with or without bLF (200 μg/mL), and analyzed by indirect immunofluorescence assay (IFA) to determine infection rates (File S1). Infection rates for DENV-1 to -4 neared 100% in the absence of bLF, but significantly decreased in the presence of bLF, especially for DENV-2, -3, and -4. In the presence of bLF, the infection rates of DENV-1 to -4 decreased by 43%, 76%, 61% and 56%, respectively, relative to rates observed in the absence of bLF. 

The anti-DENV activity of bLF was also measured by plaque reduction assay, during which the monolayer of Vero cells was infected with approximately 200 PFU DENV-1 to -4 with or without bLF (200 μg/mL). In the presence of bLF, DENV-1 to -4 plaque numbers were decreased by 41%, 81%, 41% and 43%, respectively. The half maximal inhibitory concentrations (IC_50_) of bLF were estimated and listed in Table 1. The IC_50_ of bLF was 165.8, 40.7, 166.7, and 164.5 μg/mL for DENV1-4, respectively. Taken together, the IFA and plaque reduction assay results suggest that bLF exhibits antiviral activity against four serotypes of DENV. Bovine LF appeared to inhibit most substantially DENV-2 infection; thus, DENV-2 was selected for use in the following experiments to study the potential mechanism by which bLF inhibits DENV infection.

### 2.2. Steps of DENV-2 Infection Inhibited by bLF

**Figure 1:**
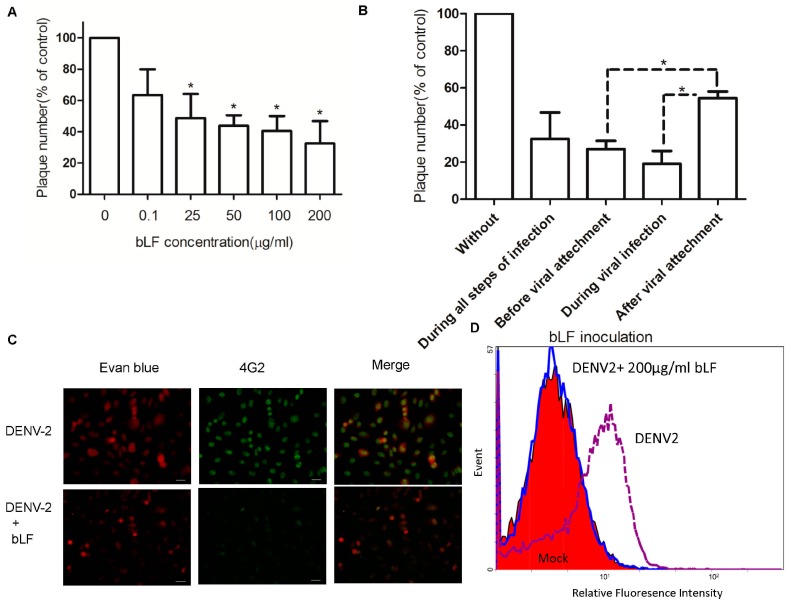
Steps of DENV-2 infection inhibited by bLF. Vero cells were pretreated with various concentrations of bLF (0, 0.1, 25, 50, 100, 200 μg/mL), then lactoferrin (LF)-treated cells were infected with DENV-2 and bLF mixture for 1.5 h at 37 °C. Subsequently, the cells in each well were overlaid with 4 mL of Dulbecco’s Modified Eagle Medium (DMEM) containing methyl cellulose supplemented with various concentrations of bLF and incubated for 7 days at 37 °C. The plaque number was counted and compared with the virus control (* *p* < 0.05). Data are mean ± SD of three duplicates. (**A**) In order to determine the steps of DENV-2 infection inhibited by bLF, 200 μg/mL bLF was added at different stages of virus infection, including during all steps of infection, before viral attachment (pre-treatment), during viral infection (co-treatment), and after viral attachment (post-attachment, DENV-2-bound cells were incubated with bLF for 1 h at 37 °C); and plaque number was counted and compared (* *p* < 0.05); (**B**) To examine whether bLF inhibits DENV-2 binding to the cellular membrane. Vero cell monolayers were treated with 200 μg/mL bLF at 4 °C for 1 h, then incubated with DENV-2 at an MOI of 5 at 4 °C for 1 h. After three washes with ice-cold phosphate-buffered saline (PBS), the cells were incubated at 37 °C for 48 h, and then analyzed by immunofluorescence assay (IFA); (**C**) The bLF-treated cells incubated with DENV-2 at a multiplicity of infection (MOI) of 50 at 4 °C for 1 h, fixed immediately after three washes with ice-cold PBS, and then analyzed by flow cytometry; scale bar: 100 μm (**D**) Using 4G2 MAb and HRP-conjugated goat anti-mouse antibody.

In order to study the inhibitory effect of bLF on DENV-2 infection, Vero cells were treated with various concentrations of bLF (0, 0.1, 25, 50, 100, 200 μg/mL) at different stages of virus infection, including during all stages of infection (full-course treatment) (Figure 1A,B), before viral attachment (pre-treatment) (Figure 1B), during viral infection (co-treatment) (Figure 1B), and after viral attachment (post-attachment) (Figure 1B). No cytopathic effect was observed in the control cells treated with the same concentration of bLF (data not shown). When bLF was present in concentrations of 25 μg/mL and greater during the full course of DENV-2 infection, plaque numbers significantly decreased relative to the bLF-free control (Figure 1A).

Among the 200 µg/mL treatment groups, the reductions in DENV-2 plaque number were more substantial when bLF was added during all steps of infection (67%), prior to viral attachment (73%), or during viral infection (82%), compared with after viral attachment (47%) (Figure 1B) (*p* < 0.05). No inhibitory effects were observed against DENV-2 infection when the viruses were pre-incubated with bLF (data not shown). These results indicate that bLF blocks DENV-2 infection during early steps of viral entry, especially during the binding of virus to cellular membrane. 

### 2.3. Bovine LF Inhibits DENV-2 Binding to Cellular Membrane

To examine the effect of bLF on DENV-2 binding to the cellular membrane, a monolayer of Vero cells was treated with 200 μg/mL bLF at 4 °C for 1 h, then incubated with DENV-2 at 4 °C for 1 h. After three washes with ice-cold PBS, the cells were analyzed by IFA (at an MOI of 5 and after being incubated at 37 °C for 48 h) (Figure 1C) and flow cytometry (at an MOI of 50 and fixed immediately) (Figure 1D). Infection in the presence of bLF produced significantly fewer IFA-positive cells (59%) than infection in the absence of LF (100%) (*p* < 0.05) (Figure 1C). Additionally, the flow cytometry analysis indicates that bLF significantly inhibited the binding of DENV-2 to the cellular membrane (6.4% of cells positive for DENV-2) compared with the binding that occurred in the absence of bLF (58.9%) (*p* < 0.05) (Figure 1D). These results indicate that bLF blocks the binding of DENV-2 to the cellular membrane.

### 2.4. Role of HS in Anti-DENV-2 Activity of bLF

**Figure 2:**
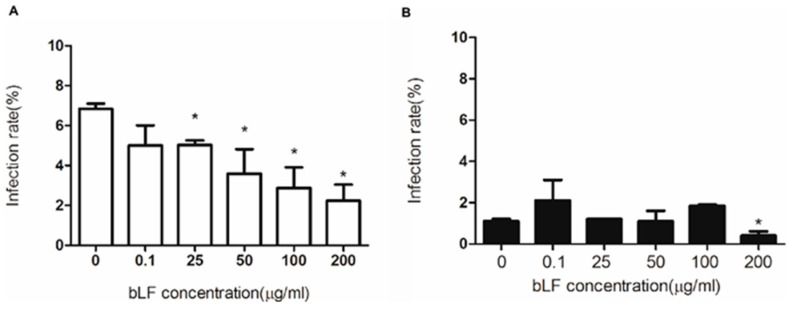
Role of heparan sulfate (HS) in the anti-DENV-2 activity of bLF. The HS-expressing CHO-K1 (**A**) and HS-deficient CHO-pgsA745 (**B**) cells were pretreated with various concentrations of bLF for 1 hour at 37 °C, then infected with DENV-2 at MOI of 5 for 1 h at 37 °C, and analyzed by infectious center assay. The infection rate equals 100 × (plaque number/cell number) (* *p* < 0.05). Data are mean ± SD of three duplicates.

HS is a possible receptor for DENV-2 and is a bLF-binding target on the cellular membrane [11,18]. Thus, to determine the role of HS in the anti-DENV-2 activity of bLF, we treated HS-expressing Chinese hamster ovary (CHO)-K1 and HS-deficient CHO-pgsA745 cells with various concentrations of bLF, then infected the cells with DENV-2 at MOI of 5 (Figure 2). The infectious center assay indicates that in the absence of bLF, the infection rate of CHO-K1 cells (Figure 2A) was significantly higher than that of CHO-pgsA745 cells (Figure 2B), which suggests that HS plays a significant role in DENV-2 infection. The infection rates of HS-expressing CHO-K1 cells significantly decreased in a dose-dependent manner in the presence of bLF (Figure 2A). However, the infection rates of HS-deficient CHO-pgsA745 cells were not influenced by the presence of bLF (Figure 2B). These results suggest that the expression of HS plays a role in the inhibition of DENV-2 infection by bLF.

### 2.5. Role of DC-SIGN in Anti-DENV-2 Activity of bLF

**Figure 3:**
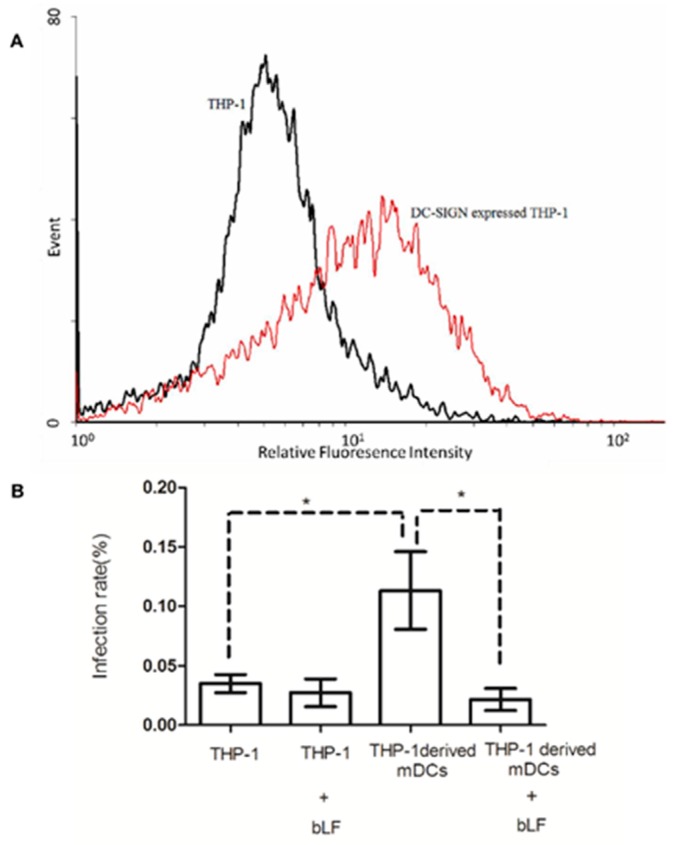
Role of DC-SIGN in the anti-DENV2 activity of bLF. The THP-1 cells were stimulated with recombinant IL-4, GM-CSF, and TNF-α according to a previous report [19], and dendritic cell-specific intercellular adhesion molecule 3-grabbing non-integrin (DC-SIGN) expression was subsequently measured by flow cytometry using mouse anti-human DC-SIGN antibodies and fluorescein isothiocyanate (FITC)-conjugated goat anti-mouse immunoglobulin G (IgG) antibodies (**A**) The DC-SIGN-expressing and non-expessing THP-1 cells were treated with 200 μg/mL bLF, then infected with DENV-2 at MOI of 5, and analyzed by infectious center assay; (**B**) The infection rate equals 100 × (plaque number/cell number) (* *p* < 0.05).

DC-SIGN is a bLF-binding protein that is expressed on the surface of dendritic cells, and a possible receptor for DENV infection [7,12]. To investigate the role that DC-SIGN plays in the anti-DENV-2 activity of bLF, THP-1-derived and DC-SIGN-expressing dendritic cells were stimulated with recombinant IL-4, GM-CSF, and TNF-α according to a previous report [19], and DC-SIGN expression was estimated by flow cytometry (Figure 3A). The fluorescence intensity indicated that DC-SIGN expressed on the stimulated THP-1 cells. The DC-SIGN-expressing and non-expressing THP-1 cells were first treated with 200 μg/mL bLF, then infected with DENV-2 at a MOI of 5, and analyzed by infectious center assay (Figure 3B). The results showed that in the absence of bLF, the infection rate of DENV-2 in DC-SIGN-expressing THP-1 cells was significantly higher than that in THP-1 cells not expressing DC-SIGN (*p* < 0.05), which suggests that DC-SIGN plays a role in DENV-2 infection. The infection rate of DC-SIGN-expressing THP-1 cells significantly decreased in the presence of bLF (*p* < 0.05). However, the infection rates of bLF-treated or untreated DC-SIGN-nonexpressing THP-1 cells were similar to one another. These results suggest that bLF may interact with DC-SIGN to reduce susceptibility of DC-SIGN-expressing cells for the DENV-2 infection.

### 2.6. Role of LDLR in Anti-DENV-2 Activity of bLF

**Figure 4:**
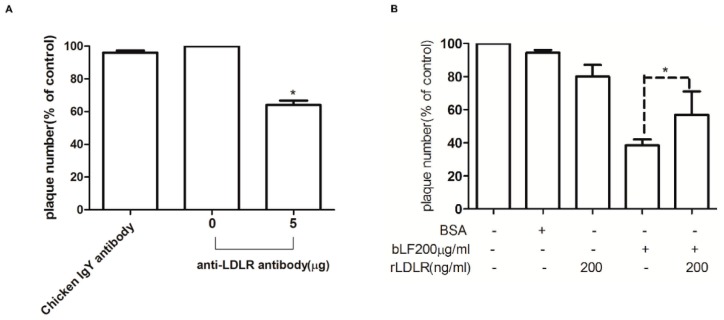
The role of low-density lipoprotein receptors (LDLR) in bLF inhibition of DENV-2 infection. Vero cells were incubated with anti-LDLR antibody, then infected with 200 PFU of DENV-2. (**A**) Vero cells were treated with bLF and recombinant LDLR alone or together; then infected with 200 PFU of DENV-2; (**B**), and analyzed in a plaque reduction assay (* *p* < 0.05).

Our previous report has shown that bLF inhibits JEV infection by binding to LDLR [6]. Thus, we firstly determined the role of LDLR in DENV-2 infection. A monolayer of Vero cells was incubated with 5 μg anti-LDLR antibody at 4 °C for 1 h, infected with DENV-2, and then virus production was estimated by plaque assay (Figure 4A). The results indicate that the anti-LDLR antibody significantly blocks DENV-2 infection by up to 36% compared with the virus control. This result suggests that LDLR might be involved in DENV-2 entry into cells.

Then, to test the role of bLF in blocking the interaction between LDLR and DENV-2, recombinant LDLR (rLDLR) was used in a plaque reduction assay to attenuate the inhibitory effect of bLF on DENV-2 infection (Figure 4B). A Vero cell monolayer was incubated with a mixture of 200 μg/mL bLF and/or 200 ng/mL rLDLR, and then infected with approximately 200 PFU of DENV-2. The effect of rLDLR on DENV-2 infectivity was estimated by the number of plaque formation. The plaque formation of DENV-2 infection significantly decreased in Vero cells treated with only 200 ng/mL rLDLR (20% inhibition) or 200 μg/mL bLF (62% inhibition). However, the inhibitory effect of bLF was attenuated in the presence of rLDLR. The plaque count increased significantly from 38% without rLDLR to 58% with 200 ng/mL rLDLR (*p* < 0.05). These results indicate that bLF may interact with LDLR, and that rLDLR attenuates the inhibitory effect of bLF against DENV-2 by competing with cellular LDLR.

### 2.7. Mouse Challenge Experiments

**Figure 5:**
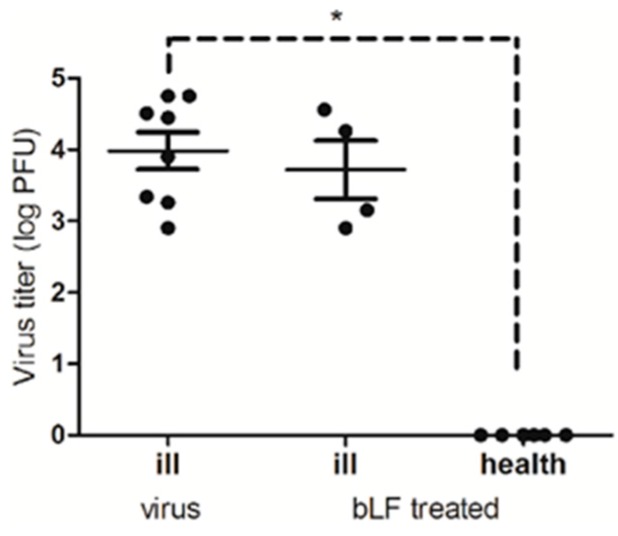
The anti-DENV-2 activity of bLF in suckling mice. One-day old suckling mice were inoculated intracranially with 1 × 10^4^ PFU of DENV-2 or a mixture of DENV-2 and 200 μg bLF by intracranial (i.c.) injection. These mice were sacrificed at 13 days post-inoculation, and the brain was collected for virus titer determination by plaque assay.

In order to study the anti-DENV-2 activity of bLF in vivo, suckling mice were inoculated intracranially with 1 × 10^4^ PFU (25-fold LD_50_) of DENV-2, or a mixture of DENV-2 and 200 μg bLF. According to the previous report, DENV-2 infected mice died around 15 days post-inoculation [20]. In this study, we recorded morbidity up to 13 days post-inoculation for both experimental groups, then euthanized all experimental mice and determined the virus titer from collected brain tissues (Figure 5). All of the mice inoculated with DENV-2 alone developed signs of illness, including dehydration, paralysis, and a lack of interest in suckling. Virus titers from the brains ranged from 10^2.9^ to 10^4.75^ PFU/per brain, averaging 10^4.47^ PFU/per brain DENV-2 for these mice. Only four of the 10 mice inoculated with premixed DENV-2 and bLF showed signs of illness. The brains of these four mice had virus titers ranging from 10^2.9^ to 10^4.56^ PFU/per brain, with an average of 10^3.75^ PFU/per brain. Six mice in this group remained healthy, and their brains produced virus titers below the detection limit (p < 0.05 compared to virus-only inoculated mice). These in vivo experiments demonstrate that bLF can inhibit DENV-2 infection and decrease morbidity by 60% compared with an untreated group.

## 3. Discussion

Bovine LF exhibits antiviral activities by binding to and interacting with viral particles, such as hepatitis C virus, rotavirus, poliovirus, echovirus, enterovirus [3,21,22], or with viral receptors on the cellular membrane, as in the cases of poliovirus, hepatitis B virus, and enterovirus [3,21,23]. In addition, we and other research groups demonstrated that bLF reduces infection of arboviruses such as Sindbis virus, Semliki Forest virus, Toscana virus, and JEV by binding membrane-bound viral receptor candidate(s) [6,16,24]. In the present study, we demonstrate that bLF prevents DENV infection by interfering with the virus entry machinery.

Multiple steps are involved in DENV entry into cells, including receptor binding, endocytosis, penetration, fusion, and uncoating 1 [10]. Many reports have shown that the bLF blocks virus infection primarily at early steps of virus entry [1]. Here, our results show that the presence of bLF prior to or during virus infection reduces virus yield and exhibits significant anti-DENV-2 activity. These results are consistent with our previous JEV study showing that bLF inhibits dengue virus infection mainly at the steps of virus attachment [6]. Also, bLF added after viral attachment inhibited DENV-2, and that might associate with the multiple enzymatic activities of internalized lactoferrin, especially RNase, [25] or with innate immunity triggered by bLF through a Toll-like receptor [26].

In order to understand the mechanism of the inhibition, the IC_50_ of bLF added at different steps of DENV-2 infection was calculated and compared with those of other arboviruses, namely JEV, ZIKV, and CHIKV [6,17] (Table 2). Overall, bLF added during virus infection was efficient to inhibit arboviruses infection; when added before virus addition, bLF significantly inhibited DENV-2, JEV, and CHIKV, but not ZIKV; and when added after virus addition, bLF significantly inhibited DENV-2, JEV, and ZIKV, but not CHIKV. It seemed that DENV-2 was more sensitive to the inhibitory effect of bLF that inhibited binding/entry and post-entry steps in the DENV-2 life cycle. The inhibitory effect of bLF at the DENV-2 post-entry step requires further investigation.

N it appears at the first toimeHS is the first molecule to have been identified as a possible receptor for DENV-2 [11]. The internalization of DENV-2 is also mediated by HS [27]. A previous study demonstrated that bLF binds electrostatically to HS via a direct interaction of negatively-charged HS and positively-charged bLF [16,28]. In this study, bLF strongly prevents DENV-2 from binding to the cellular membrane, and also inhibits DENV-2 at the penetration step. Using HS-expressing and deficient CHO cells, our results support that bLF blocks DENV-2 infection by interacting with HS on the cellular membrane. 

Bovine LF blocks the interaction between gp120 and DC-SIGN expressed on dendritic cells, and inhibits the process of transmission of human immunodeficiency virus (HIV) from dendritic cells to T cells [7]. The ion-binding region of bLF was considered important for interacting with DC-SIGN and related to its antiviral effect [7]. The mosquito-borne DENV initially infects mature human dendritic cells via engagement of receptor DC-SIGN, and viral replication occurs in DC-SIGN-expressing mature dendritic cells [12]. This study indicated that the interaction between DC-SIGN and bLF plays a role in the inhibitory effect of bLF against DENV-2 infection. 

LDLR is also a possible receptor for several viruses of the *Flaviviridae* family, such as hepatitis C virus (HCV) and JEV [6,29]. The amount of LDLR on the surface of Huh-7 cells was increased at the early stage of DENV infection [30], and LDL had showed an inhibition against DENV infection [31]. In the present study, DENV-2 infection was inhibited by chicken anti-LDLR antibody and rLDLR. These results implied that LDLR might be involved in DENV-2 entry. Our previous report indicates that bLF binds to LDLR and inhibits JEV entry into cells [6]. Here, the anti-DENV-2 effect of bLF was attenuated by the addition of rLDLR. The LDLR are responsible for regulating the extracellular cholesterol concentration; cholesterol-lowering drugs such as statin increase LDLR expression, thereby reducing the serum cholesterol level [32]. Thus, a comprehensive study on the role of LDLR in DENV-2 infection and a risk assessment study to determine the effect of serum cholesterol level and dengue infection may be required to investigate the impact of cholesterol–lowering drugs on DENV infection. 

Here, we demonstrate that bLF blocks DENV infection primarily by binding to several possible receptors for DENV-2. However, the antiviral activities of bLF were varied against DENV-1-4. This observation might reflect that some dengue serotypes may infect cells via serotype-specific mechanisms. Serotype-specific components on cellular membranes regulating the entry of the dengue virus into cells have been identified [33], namely, the 37/67-kDa high-affinity laminin receptor that has been identified as a serotype-specific receptor for DENV-1 [34]. 

Most anti-DENV agents in development bind to a specific target, such as a viral protein [35] or a molecule on or among cells [15]. Here, we demonstrate that bLF acts as an anti-DENV agent by binding to multiple specific targets that might prevent the development of a resistant virus against bLF, but this needs to be proved in a future study. Although bLF might act as a promising anti-DENV agent and orally administered bLF absorbs by intestinal receptor and appears in the blood [36], several aspects of the anti-DENV activity of bLF need to be characterized comprehensively in the future, including which lobe (N or C lobe) of the bLF molecule exerts anti-DENV activity [22], the optimal dosage and time (before or after infection) to administer bLF in vivo, and the anti-DENV activity of bLF administered orally. In addition, an additive effect has been indicated between lactoferrin and ribavirin, which is another potential anti-DENV drug [37].

## 4. Materials and Methods

### 4.1. Viruses and Cell Lines

Dengue viruses were propagated in and harvested from the supernatant of infected-C6/36 cell lines, and stored at −70 °C. Virus strains included DENV-1 Hawaii, DENV-2 16681, DENV-3 H-87, and DENV-4 H-241. Mosquito C6/36 and monkey Vero cells were cultured in minimum essential medium (MEM) (Life Technologies, Carlsbad, CA, USA) with 10% fetal bovine serum (FBS) (Biological industries, Kibbutz, Israel) at 28 or 37 °C, respectively, with 5% CO_2_. The HS-expressing CHO-K1 and HS-deficient CHO-pgsA745 cells were grown in Ham’s F12 Nutrient Mixture (GIBCO, Gaithersburg, NY, USA) with 10% FBS at 37 °C with 5% CO_2_. Human monocyte THP-1 cells were cultured in Roswell Park Memorial Institute (RPMI) medium (Life Technologies, Carlsbad, CA, USA) with 10% FBS in 37 °C with 5% CO_2_.

### 4.2. Plaque Assay and Plaque Reduction Assay

Plaque assays were used for viral titration, and plaque reduction assays to study the antiviral effect of bLF. The procedures were similar to those described in our previous report, with some modification [6]. Vero cells were seeded in six-well plates (10^5^ cells/well) and incubated at 37 °C overnight. The cultured Vero cells were infected with 200 µL of serially diluted virus for 1.5 h at 37 °C; the cell plates were rocked every 20 min during infection. Subsequently, each well was covered with 4 mL of 1.1% methyl cellulose in DMEM with 1% FBS and incubated for seven days at 37 °C. Cells were fixed with 10% formaldehyde for 40 min and stained with 0.5% crystal violet overnight. Plaque number was counted and expressed as the plaque forming unit per mL (PFU/mL).

Iron-unsaturated (apo) bLF (Sigma, St. Louis, MO, USA) (purity > 85%) was dissolved in deionized water and diluted with DMEM. In plaque reduction assays similar to the plaque assay, Vero cells were treated with various concentrations of bLF (0, 0.1, 25, 50, 100, 200 μg/mL) at different stages of virus infection, including prior to viral attachment (pre-treatment), during viral infection (co-treatment), after viral attachment (post-attachment), and at all three stages. Plaque number was counted and compared with an untreated virus control.

### 4.3. Viral binding Assay

Suspended Vero cells were harvested using 5.0 mM ethylenediaminetetraacetic acid (EDTA) in PBS. Cells (10^6^) were incubated with or without 200 μg/mL bLF at 4 °C for one hour. After washing with ice-cold PBS, bLF-treated cells were incubated with DENV-2 at a multiplicity of infection (MOI) of 50 for 1 h at 4 °C and washed three times with ice-cold PBS. The cells were fixed with 0.5% formaldehyde, and stained using the monoclonal antibody 4G2 and fluorescein isothiocyanate (FITC)-conjugated goat anti-mouse at 4 °C for one hour for each procedure. After washing, the membrane-bound DENV-2 was detected and analyzed via flow cytometer (Beckman, Porterville, CA, USA). 

### 4.4. Infectious Center Assay

An infectious center assay was used to study the role of HS in the anti-DENV-2 activity of bLF described in the previous report [6]. The HS-expressing CHO-K1 and HS-deficient CHO-pgsA745 cells were harvested from cultured flasks via treatment with 5 mM EDTA in PBS. Cells (10^6^) were mixed with 200 μL/mL bLF for 1 h at 37 °C. After centrifugation and a PBS wash, LF-treated cells were infected with DENV-2 at MOI of 5 for 1 h at 37 °C, and washed three times with PBS. The infected cells were serially diluted ten-fold and seeded on a monolayer of Vero cells in six-well plates at 37 °C for one hour. Then, each well was covered by 4 mL 1.1% methyl cellulose in DMEM with 1% FBS, and incubated at 37 °C for seven days. Cells were then fixed using 10% formaldehyde for 40 min, and stained with 0.5% crystal violet overnight. Plaques were counted and expressed as infectious rate (infection rate = plaques per well/cells per well).

### 4.5. Blocking Experiments to Investigate the Role of LDLR 

Blocking experiments were used to study further the role of LDLR on DENV-2 infection described in a previous report [6]. Vero cell monolayers in six-well plates were incubated with 5 μg/well of chicken anti-LDLR antibody (Millipore, Billerica, MA, USA) at 37 °C for one hour. Following a wash, the treated cells were infected with approximately 200 PFU of DENV-2 for one hour, and a plaque assay was carried out as described above. 

We also treated Vero cell monolayers in six-well plates with recombinant human LDLR at 200 ng/mL (R&D systems, Inc., Minneapolis, MN, USA) and bLF at 200 µg/mL along or together for one hour at 37 °C. After a PBS wash, the bLF and rLDLR-treated cells were infected with 200 PFU of DENV-2 in order to study the attenuation of rLDLR on anti-DENV-2 activity of bLF. The antiviral effects of the treatments were estimated in a protocol similar to the plaque reduction assay. 

### 4.6. Determing the Role of DC-SIGN 

The expression of DC-SIGN is inducible in human monocyte-derived THP-1 cells. The THP-1 cells were cultured with recombinant IL-4 (3.0 µg/mL), GM-CSF (1.5 μg/mL), TNF-α (0.3 μg/mL) and ionomycin (3 μg/mL) (all recombinant proteins from PEPROTECH, Rehovot, Israel), in RPMI medium with FBS, according to a previous report [19]. The culture medium was changed on the third and fifth days. After seven days, the cells were fixed and stained with mouse anti-human DC-SIGN (AbD Serotec, Oxford, UK) and FITC-conjugated goat anti-mouse IgG (Invitrogen, Grand Island, NY USA). The expression of DC-SIGN on THP-1 cells was detected and analyzed via flow cytometer (Beckmen, Los Olivos, CA, USA). The DC-SIGN-expressing and non-expressing THP-1 cells were used to study the role of DC-SIGN in the anti-DENV-2 activity of bLF in an infectious center assay.

### 4.7. Indirect Immunofluorescence Assay 

Vero cells were seeded in the wells of a chamber slide (Millipore, Billerica, MA, USA), cultured at 37 °C overnight, and incubated with a mixture of DENV-2 and bLF at 37 °C for one hour. After one day, the cells were fixed with 4% paraformaldehyde-PBS at room temperature for 20 min and then washed with PBS. After blocking with 3% BSA-PBS at 37 °C for one hour, the cells were stained with anti-DENV-2 mouse hyperimmune ascitic fluid (MHIAF), followed by FITC-conjugated goat anti-mouse IgG (KPL, Gaithersburg, MD, USA) in 1% Evans blue. The images were viewed using an OLYMPUS CKX41. The infection rate (infection rate = total IFA-positive cells/total counted cells) was estimated by counting positive cells under three independent fields (>100 cells/field).

### 4.8. Mouse Challenge Experiments 

The mouse experiment protocol was approved by the Committee on the Ethics of Animal Experiments of National Chung Hsing University (Approval No: 97-76, Date: 24 December 2008), following guidelines from the care and use manual of the National Laboratory Animal Center, Taiwan. Efforts were made to minimize suffering, and the mice were euthanized with 50% CO_2_ and cervical dislocation. Pregnant BALB/c mice were purchased from the National Laboratory Animal Center, National Science Council, Taiwan. One-day-old suckling mice were inoculated with 20 µL of 25 LD_50_ (1 × 10^4^ PFU) of DENV-2 (16681) or pre-mixed DENV-2 (25 LD_50_) with 200 mg bLF by intracranial (i.c.) injection. Signs of illness, including dehydration, hind leg paralysis, and a lack of interest in suckling were observed daily and recorded. These mice were sacrificed at 13 days post-inoculation and the brains were collected for virus titer determination by plaque assay.

### 4.9. Statistical Analysis

Student’s two-tailed t-test and one-way ANOVA were respectively used to analyze the differences between two groups, and multiple groups by GraphPad Prism v5.01. *p* < 0.05 was indicated as a statistically difference.

## Figures and Tables

**Table 1:** Half maximal inhibitory concentration (IC_50_) of bLF added during the whole virus infection procedure.

Virus	IC_50_ of bLF (μg/mL)	Reference
DENV-1	165.8 ± 35.1	This study
DENV-2	40.7 ± 8.6	This study
DENV-3	166.7 ± 30.6	This study
DENV-4	164.5 ± 41.0	This study
JEV	518.3 ± 115.8	[6]
ZIKV	~200 ± 5.0	[17]
CHIKV	~400 ± 6.0	[17]

Bovine lactoferrin, bLF; Dengue virus, DENV; Japanese encephalitis virus, JEV; Zika virus, ZIKV; Chikungunya virus, CHIKV; Values are expressed as mean ± standard deviation.

**Table 2:** Half maximal inhibitory concentration (IC_50_) of bLF added at different steps of virus infection.

Virus	IC_50_ of bLF Added at Different Steps of Virus Infection(μg/mL)			Reference
	Before Virus Addition	During Virus Addition	After Virus Addition	
DENV2	20.8	18.0	74.9	This study
JEV	385.6	ND	228.9	[6]
ZIKV	>1000	<1000	<1000	[17]
CHIKV	~1000	<1000	>1000	[17]

Not determined, ND.

## Supplementary Materials

Supplementary materials can be found at www.mdpi.com/1422-0067/18/9/1957/s1.

## Abbreviations

bLF	Bovine lactoferrin
DENV	Dengue virus
HS	Heparan sulfate
DC-SIGN	Dendritic cell-specific intercellular adhesion molecule 3-grabbing non-integrin
LDLR	Low-density lipoprotein receptors
