# Supplementary material for: Bovine Lactoferrin Inhibits Dengue Virus Infectivity by Interacting with Heparan Sulfate, Low-Density Lipoprotein Receptor, and DC-SIGN
Source: Int J Mol Sci. 2017 Sep 12;18(9):1957. doi: 10.3390/ijms18091957 (PMC5618606; doi:10.3390/ijms18091957)
Supplement: Supplementary file 1 [file ijms-18-01957-s001.pdf]

Supplementary File 1

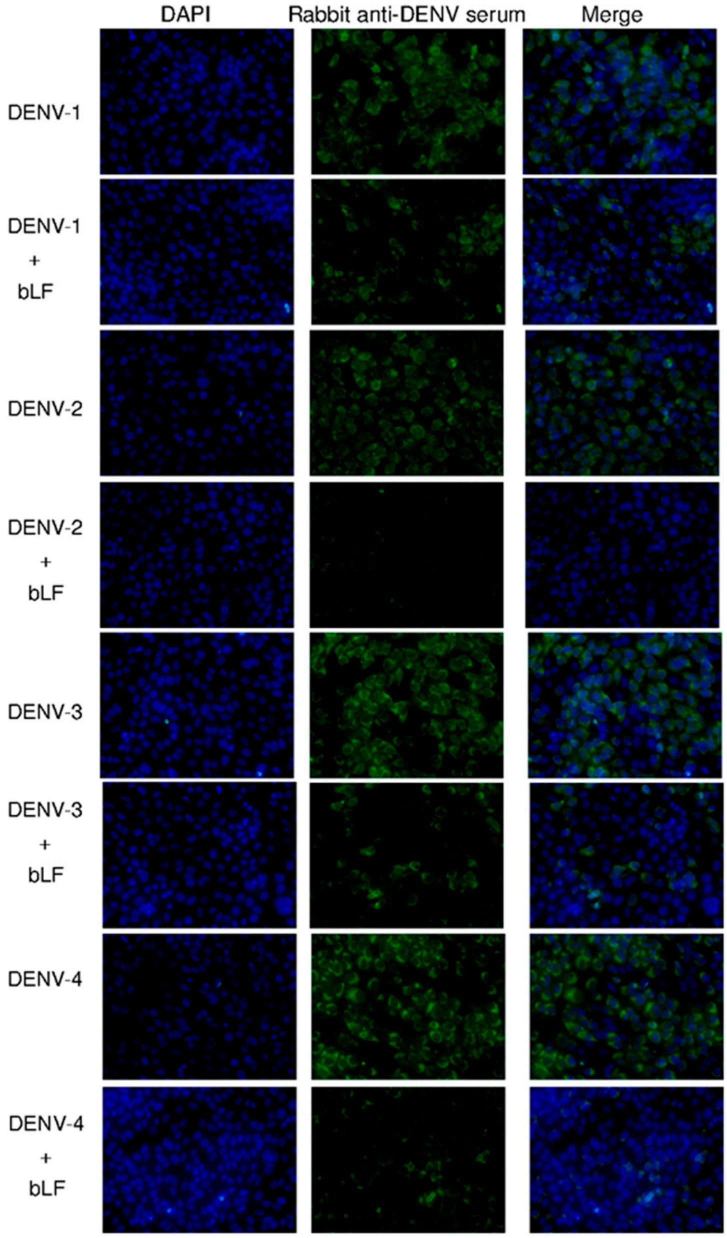

Supplementary Figure 1. Inhibitory effect of bLF against DENV-1-4 infection. Vero cell monolayers were infected with DENV1-4 at a MOI of 5, with or without bLF (200 μg/ml). After 24 hours, the cells were fixed and stained with DAPI and rabbit anti-DENV-1-4 antibodies, followed by goat anti-rabbit Alexa Fluor 488-conjugated secondary antibody. Images were observed and captured by fluorescent microscope.
